# Choledochal cyst and aberrant biliary configuration along with situs inversus totalis: a case report

**DOI:** 10.11604/pamj.2021.38.398.29228

**Published:** 2021-04-23

**Authors:** David Eng Yeow Gan, Dinesh Alagoo, Kheng Hooi Chan, Rebecca Xin Yee Choi, Harivinthan Sellappan, Mohd Sharifudin Sharif, Firdaus Hayati

**Affiliations:** 1 Department of Surgery, Queen Elizabeth Hospital, Ministry of Health Malaysia, Kota Kinabalu, Sabah, Malaysia,; 2 Department of Surgery, Faculty of Medicine, Universiti Kebangsaan Malaysia Medical Centre, Cheras, Kuala Lumpur, Malaysia,; 3 Gleneagles Kota Kinabalu, Kota Kinabalu, Sabah, Malaysia,; 4 Department of Surgery, Faculty of Medicine and Health Sciences, Universiti Malaysia Sabah, Kota Kinabalu, Sabah, Malaysia

**Keywords:** Choledochal cyst, situs inversus, case report

## Abstract

Situs inversus totalis is the complete transpositioning of thoracoabdominal viscera into a mirror image of the normal configuration. Choledochal cyst is the congenital cystic dilation of the biliary tract. Both these conditions coexisting in a patient is extremely rare. We hereby present a case of type IC choledochal cyst in a patient with situs inversus totalis presenting with biliary sepsis secondary to choledocholithiasis. Also detailed are the management and operative strategies employed to deal with this rare entity.

## Introduction

*Situs inversus totalis* (SIT) or complete mirroring of the heart and viscera, is the opposite to a normal human body configuration or situs solitus. It is a rare benign condition with incidence of about 1: 4000 to 1: 8000 individuals [1]. Choledochal cyst (CC) on the other hand is described as the congenital cystic dilatation of the biliary tree and is a rarer condition than SIT with an incidence of 1 in 100,000 to 150,000 in Western populations and up to 1: 1000 in Asian populations, particularly Japan [2]. The coexistence of these two conditions is extremely rare with little literature to describe it. We hereby report a case of a patient with SIT and CC presenting with biliary sepsis.

## Patient and observation

Our patient is a 42-year-old female immigrant of Bajau descent with no prior history of medical illnesses who presented to a district facility with a short history of epigastric pain, fever with chills and rigors; as well as multiple bouts of vomiting for 3 days. She did not notice any pale stools or tea-coloured urine and neither did she complain of any respiratory symptoms suggestive of Kartagener syndrome. Physical examination noted apex beat on the right whereas the liver was not palpable. Serum bilirubin was raised at 101.5 umol/L. A peripheral blood film also revealed concomitant malarial infection with the parasite *Plasmodium knowlesi*. Chest and abdominal radiographs were consistent with dextrocardia and situs inversus. She was septic and hypotension despite fluid resuscitation requiring inotropic support. She was started on intravenous Ceftriaxone in suspicion of biliary sepsis, as well as Artesunate and Riamet (Artemether/ Lumefantrine) for malaria.

**Figure 1:**
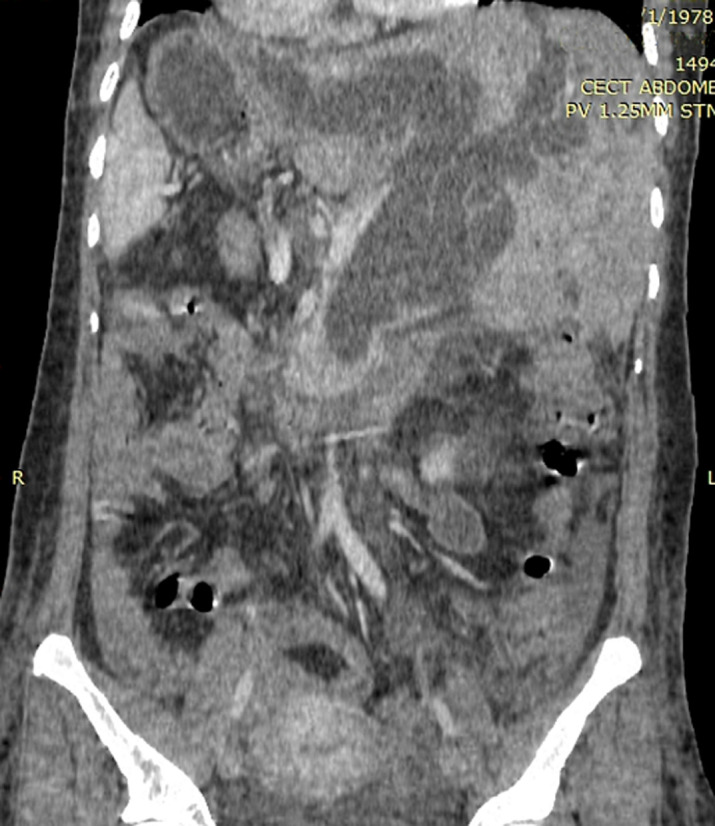
computed tomography revealed a fusiform dilatation of the extrahepatic and intrahepatic ducts with the CBD

Our patient was then transferred to a regional centre where ultrasonography and computed tomography was performed. Findings revealed fusiform dilatation of the extrahepatic and intrahepatic ducts with the common bile duct (CBD) measuring 28 mm in widest diameter (Figure 1). Also noted was a large stone in the common bile duct measuring 25 mm. Cystic duct appeared to insert into the right (left sided) hepatic duct. There were no stones in the gallbladder or intrahepatic ducts. Apart from the mirror-image orientation, abdominal vasculature was grossly normal with the common hepatic artery arising from the celiac trunk and right hepatic artery traversing posterior to the CBD. Inferior vena cava was on the patient´s left and portal vein arose behind the neck of the pancreas. Her cardiac function and valvular anatomy were normal on echocardiogram. She was then transferred to our centre where ERCP was performed with biliary stenting while awaiting surgery.

**Figure 2:**
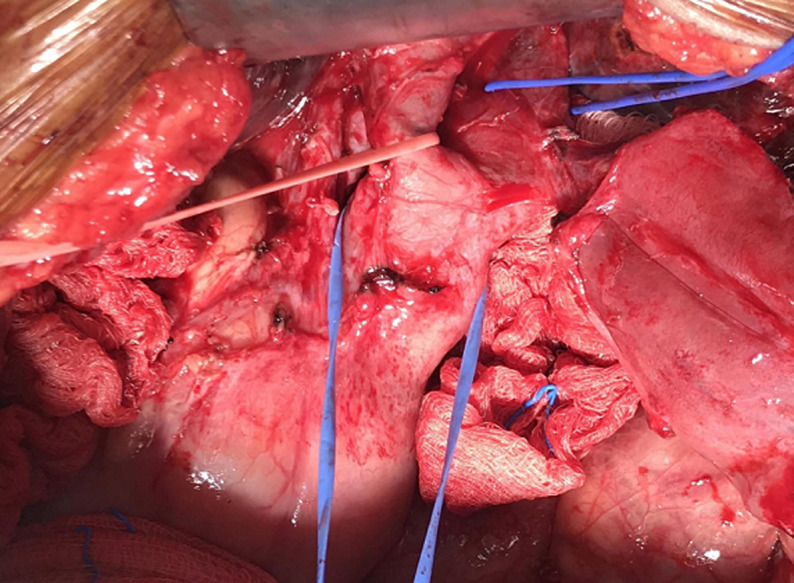
cystic dilation of the extrahepatic biliary tract with low bifurcation of the CBD

**Figure 3:**
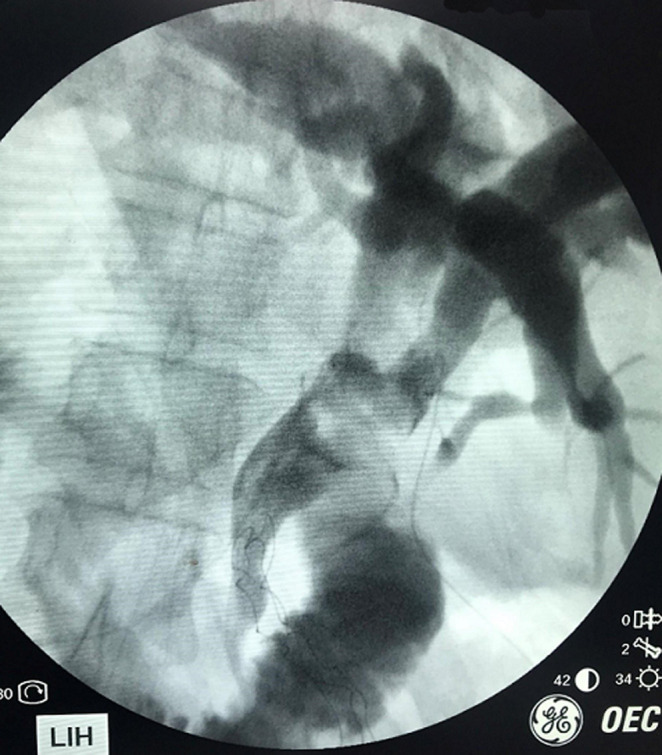
operative cholangiogram showing fusiform dilation of intra and extrahepatic bile ducts with a calculus in the common bile duct; feeding tube is seen entering the right hepatic duct (left in this case) via cystic duct

**Figure 4:**
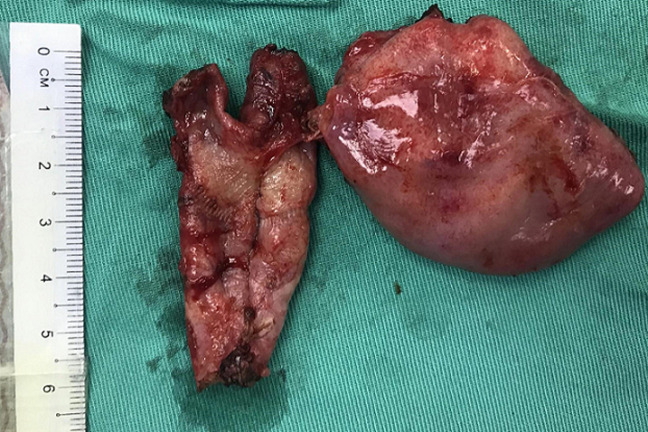
resected specimen demonstrating insertion of the cystic duct into the right hepatic duct

We performed a cholecystectomy, choledochectomy and Roux-en-Y hepatico jejunostomy 2 weeks later. A laparoscopic approach was initially attempted but abandoned in view of dense omental adhesions to the gallbladder and liver. Our open approach involved a left subcostal incision with the surgeon standing on the patient´s left. Self-retaining retractors were deployed to aid retraction of the abdominal wall, liver and viscera. A fundus-first cholecystectomy was performed followed by a cholangiogram to clearly delineate the biliary tree. Fusiform dilation of extrahepatic bile ducts was noted, consistent with a Todani type 1C choledochal cyst. There was also low bifurcation of the CBD below the insertion of the cystic duct which was into the right (left sided) hepatic duct (Figure 2). No anomalous biliopancreatic junction was seen in the cholangiogram (Figure 3). Choledoscopy of the biliary tract revealed no abnormal mucosa or stricture. Choledochectomy was performed proximally at extrahepatic portion of right and left hepatic ducts, and distally at the intrapancreatic portion. A reverse Kocher manoeuvre was performed for mobilization of the duodenum and pancreatic head. The right and left hepatic ducts were sutured together to form a common channel followed by reconstruction with a Roux-en-Y hepaticojejunostomy. The resected specimen demonstrated the insertion of the cystic duct into the right hepatic duct (Figure 4). She recovered well post operatively and was discharged on day 10. Upon follow up, she was well without any complications.

## Discussion

SIT and CC are rare entities themselves let alone occurring together. To our knowledge this is the third such case reported, the second to receive surgical treatment. The first case described was a type IC CC noted during cholecystectomy for cholecystitis, but the patient was unfortunately lost to follow up before definitive surgery. The second was discovered during investigation for cholangitis. Also a type I CC, it was successfully treated with excision of the cyst with Roux-en-Y hepaticojejunostomy. Cholangiographic and operative data from both cases demonstrated no biliary tree anatomical anomalies [3, 4]. As with our case, the lead surgeon remained at the patient´s left side throughout surgery. Self-retaining retractors aided significantly in retraction of the abdominal viscera whereas the first assistant actively aided dissection and fine retraction of the deep tissue.

A normal biliary tree is observed in just over 50% of the population. Much of the literature detailing biliary tree variations focus on right and left intrahepatic duct variations, classified according to the Huang and Cho classification respectively [5]. Congenital extrahepatic biliary duplications on the other hand are classified according to the Choi classification [6]. However, there is no system to classify biliary variants that do not fit into this criterion. A similar case of low CBD bifurcation has been reported by Boutros *et al*. in a situs solitus patient [7]. Despite the lack of a system to classify cystic duct anomalies, they have been widely reported, with the cystic duct draining into the left hepatic duct in less than 0.5% of patients observed [8]. In this anomaly, the right hepatic duct is at risk of injury if mistaken for the cystic duct. There is however more robust literature regarding SIT patients undergoing pancreatoduodenectomy for various malignancies, although no association has been proven thus far. Surgery for SIT patients with pancreatobiliary malignancy has been documented since 1985 [9]. Various anomalies have been reported, mainly involving the hepatic vasculature, portal vein and its tributaries; as well as polysplenia. Most authors advocate a detailed preoperative radiological mapping of the biliary tree and abdominal vasculature and careful dissection of the portal structures [1, 10-12].

Type I choledochal cyst is the most common (50-90%) followed by type IV (10-40%) with both types displaying the highest risk of developing malignancy. Thus, complete excision of the CC is advocated with some authors even suggesting the need for liver resection and liver transplant for extensive intrahepatic biliary involvement [13]. Lal *et al*. described the treatment of 33 patients with type IV CC by excision of the extrahepatic portion with a wide bilio-enteric anastomosis [14]. Close surveillance is required to detect complications arising from the intrahepatic CC like strictures, hepatolithiasis and malignancy.

## Conclusion

In conclusion, SIT is not a contraindication to hepatobiliary surgery. Therefore, when encountering such patient, thorough preoperative mapping of the biliary system and periportal vasculature is crucial. Meticulous dissection technique and a consistent awareness regarding the mirrored orientation of abdominal viscera serve to avert iatrogenic injuries when confronting such cases.
